# Les deux complications majeures du tabac en une seule image!

**DOI:** 10.11604/pamj.2018.30.252.16393

**Published:** 2018-08-06

**Authors:** Hanane Asri, Adil Zegmout

**Affiliations:** 1 Service de Pneumologie de l'Hôpital Militaire d'Instruction Mohammed V, Faculté de Médecine et de Pharmacie de Rabat, Université Mohammed V, Rabat, Maroc

**Keywords:** Tabagisme, cancer, emphysème pulmonaire, Smoking, cancer, lung emphysema

## Abstract

Smoking is the leading cause of preventable death worldwide, it is responsible for 90% of bronchopulmonary cancers and is the main cause of chronic bronchitis and emphysema, two disorders which contribute to chronic obstructive pulmonary disease (COPD). We here report the case of a 58-year old not weaned chronic tabagic patient with a 2-month history of diffuse abdominal pain evolving in a context of alteration of the general state. Clinical examination showed generally poor health. Pleuropulmonary examination objectified reduction of vesicular breath sounds in the right hemithorax and diffuse abdominal susceptibility and massive left subclavicular lymphadenopathy. Thoraco-abdominal CT scan showed pleural, intra-abdominal and retroperitoneal tissue infiltration and diffuse bilateral lung emphysema (Figure). Bronchial fibroscopy objectified bud obstructing the orifice of the apical bronchus of the right upper lobar bronchus. Anatomopathologic study of bronchial biopsy and lymph node biopsy showed non-differentiated carcinoma. Evolution was marked by patient’s death after two weeks. This study aims to highlight fatal outcome due to these two complications due to tobacco use in the same patient in order to emphasize the importance of prevention awareness of the damages of tobacco use and on smoking cessation.

## Image en médecine

Le tabagisme est la première cause de mortalité évitable dans le monde, il est responsable de 90% de cancers bronchopulmonaire et c'est la principale cause de bronchite chronique et d'emphysème, deux troubles qui composent la maladie pulmonaire obstructive chronique (MPOC). Patient âgé de 58 ans, grand tabagique chronique non sevré qui présente depuis 2 mois des douleurs abdominales diffuses, le tout évoluant dans un contexte d'altération général. L'examen clinique a trouvé une patient en mauvais état général, pleuro pulmonaire a objectivé une diminution des murmures vésiculaires dans l'hémi champs thoracique droit, et sensibilité diffuse abdominale et énorme adénopathie sus claviculaire gauche. La tomodensitométrie thoraco-abdominale a objectivé la présence d'une infiltration tissulaire pleurale et abdominale intra et rétro péritonéale et emphysème pulmonaire diffus bilatéral (Figure). La fibroscopie bronchique a trouvé un bourgeon obstruant l'orifice de la bronche apicale de bronche lobaire supérieure droite. L'étude anatomopathologique de la biopsie bronchique et la biopsie ganglionnaire étaient en faveur d'un carcinome non différencié et l'évolution était marquée par le décès du patient après deux semaines. On rapporte à partir de cette observation l'évolution fatale par les deux complications du tabac chez le même patient d'où l'intérêt de la prévention qui repose sur la sensibilisation des dangers du tabac et sevrage tabagique.

**Figure 1:**
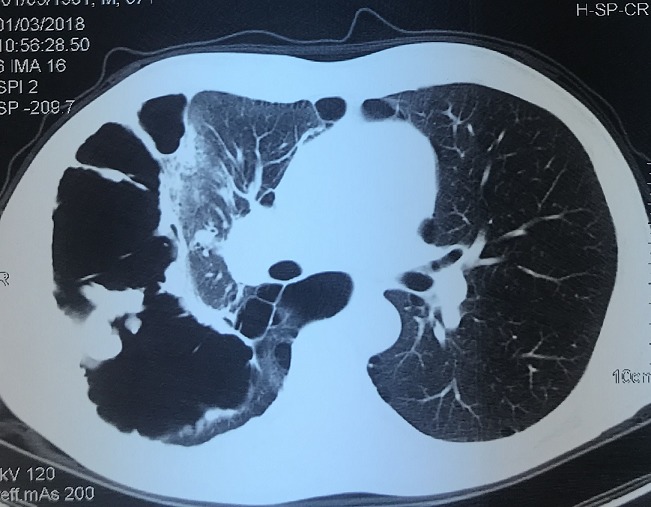
Coupe parenchymateuse de scanner thoracique objectivant une infiltration tissulaire pleurale et emphysème pulmonaire diffus

